# Alcohol and Insanity

**Published:** 1899-10

**Authors:** 


### ALCOHOL AND INSANITY.

Could the drunkard see for a moment that in his slavery to a debasing vice he may be planting the seeds of misery, of degradation, of crime, and possibly of idiocy in his future offspring, he might realize in such force the words of the inspired teacher, "to eat, drink, and be merry," to indulge in full the animal passions of his nature; "but remember for all this God shall bring you to judgment," as to cause him to start back in terror as from the brink of hell. Statistics tell with fearful force upon the story of idiocy, crime, and insanity blossoming and ripening in fruit bitter as the apples of Sodom, from seed planted in a previous generation. Statistics by one of our most painstaking physicians show that out of 300 idiots whose history could be traced, 155 were the children of drunken parents. In a large asylum, as stated by the superintendent, out of 958 admissions one-fourth could be clearly traced to intemperance in drink. This report is confirmed by the lunacy commission, who, in taking an average of the last five years proved that drink as a cause averages from one-fourth to one-fifth of all the admissions to insane hospitals. The offspring of drunken parents, says Forbes Winslow, by a law of vital physiology, if they do not actually follow in the wake of their parents, still are in great danger of exhibiting some trace of moral or mental obliquity or of a nervous disorder clearly traceable to a deterioration of physical structure, probably seated in the brain, caused by a long and persistent indulgence in the use of intoxicating liquors.—*Medical Times*, September, 1899.

